# Effects of Alkali Metal (Li, Na, and K) Incorporation in NH_2_–MIL125(Ti) on the Performance of CO_2_ Adsorption

**DOI:** 10.3390/ma12060844

**Published:** 2019-03-13

**Authors:** Lifang Song, Cheng Xue, Huiyun Xia, Shujun Qiu, Lixian Sun, Huaxin Chen

**Affiliations:** 1 School of Materials Science and Engineering, Chang’an University, Xi’an 710061, China; 18677367083@163.com (C.X.); xiahy@chd.edu.cn (H.X.); 2 School of Materials Science and Engineering, Guilin University of Electronic Technology, Guilin 541004, China; qiushujun@guet.edu.cn (S.Q.); sunlx@guet.edu.cn (L.S.)

**Keywords:** alkali metal, CO_2_ adsorption, metal-organic frameworks, NH_2_-MIL125

## Abstract

A series of titanium-based, metal–organic framework (MOF) materials, *x*M@NH_2_-MIL125(Ti) (*x* is the alkali metal loading percentage during the synthesis; M = Li, Na, K), have been synthesized solvothermally. Alkali metal doping in the NH_2_–MIL125(Ti) in situ solvothermal process demonstrated a vital modification of the material structure and surface morphology for the CO_2_ adsorption capacity at ambient conditions. By changing the reactants’ precursor, including different kinds of alkali metal, the morphology of *x*M@NH_2_–MIL125(Ti) can be adjusted from a tetragonal plate through a circular plate to a truncated octahedron. The variation of the alkali metal loading results in substantial differences in the CO_2_ adsorption. The properties of *x*M@NH_2_–MIL125(Ti) were evaluated via functional group coordination using FT-IR, phase identification based on X-ray diffraction (XRD), surface morphology through scanning electron microscopy (SEM), as well as N_2_ and CO_2_ adsorption by physical gas adsorption analysis. This work reveals a new pathway to the modification of MOF materials for high-efficiency CO_2_ adsorption.

## 1. Introduction

With rapid economic growth, excessive carbon consumption correlated with enhanced CO_2_ emission into the atmosphere has caused involved environmental problems, such as global warming and climate change [1]. Carbon capture and storage (CCS) has been considered a prospective technological strategy to slow down gas emissions and alleviate the climate [2]. Up to now, there are multifarious approaches to CO_2_ storage and separation, such as solvent absorption, physicochemical adsorption, membrane separation, cryogenic distillation, and so on [3]. Among the technologies applied for this purpose, adsorption of CO_2_ into porous solid materials, such as zeolites, mesoporous silicas, porous carbon, and metal–organic frameworks (MOFs), has been gaining increasing attention due to its low energy requirements, cost-effectiveness, high adsorption capacity, and regeneration [4,5,6,7].

MOFs, exhibiting large internal surface area, controllable porosity and pore walls, and affinity for particular valuable gases, have been regarded as potential CO_2_ adsorption and separation materials under mild conditions. Usually, MOFs are constructed through metal ions or clusters as connected centers, and polyfunctional organic ligands as connected linkers. As a matter of fact, the extended framework can be controlled by selecting the appropriate metal centers and organic linkers to obtain the desired structural features and physicochemical properties. In order to promote the CO_2_ adsorption capacity and the separation selectivity over other gases, various strategies have been reported, such as metal cation incorporation [8,9], pore size and shape tuning [10], and ligand functionality [11,12,13].

The alkali metal incorporation strategy has been widely utilized in the field of hydrogen storage, which has been confirmed to be effective. However, the influence of alkali metal incorporation into MOF materials on CO_2_ capture has been relatively less investigated. Lan et al. and Cao et al. showed clearly, by multiscale simulation, that the CO_2_ uptake of lithium-doped covalent organic frameworks (COFs) was enhanced by four to eight times compared with the pristine COFs at 298 K and 1 bar [14,15]. Mu et al. studied the effect of different metal doping on the gas adsorption performance of MOFs through a grand canonical Monte Carlo (GCMC) simulation [16]. They found that CO_2_ preferentially adsorbs at the alkali metal doping position. Babarao et al. studied Mg-IRMOF-1 and Be-IRMOF-1(Isoreticular metal-organic frameworks, IRMOF), indicating that alkaline earth metal ion substitution improves adsorption capacity [17]. Xiang et al. analyzed the CO_2_ adsorption results of Li@Cu_3_(BTC)_2_, CNT@Cu_3_(BTC)_2_, and Li@CNT@Cu_3_(BTC)_2_, and they found that lithium doping actually improved CO_2_ adsorption capacity, and increased by 305% for Li@CNT@Cu_3_(BTC)_2_ [18].

Doping metal ions into an MOF can improve adsorption performance, as metal ions can increase the interaction energy between CO_2_ and MOFs. Virtually most of the reports on alkali metal incorporation in MOFs are established on theoretical models and computer simulations. Actually, direct verification of this effect through lab experiments is still a challenge. Surveying the effect of alkali metal doping in MOFs on CO_2_ capture became urgent for us. The in situ incorporation procedure of metal ions into MOFs during the solvothermal process is straightforward compared with the post-synthetic modification procedure. 

To further improve the CO_2_ adsorption performance of MOFs, the introduction of amino groups (–NR_2_) into the organic linkers are considered to be effective. The amino group can act as a Lewis basic active site, with strong binding to carbon dioxide, and the presence of the lone electron pair enhances the affinity between an MOF and carbon dioxide molecules. Zhu et al. indicated that the basic sites of MOFs can be generated by either direct synthesis or post-synthetic modification. In the case of direct synthesis, the basicity may originate from metal centers, organic ligands, and the interplay between the two. For post-synthetic modification, both metal sites and ligands are available for the introduction of basic species [19]. Kim et al. point out that pore engineering of MOFs utilizing Lewis basic functionalities are beneficial for CO_2_ adsorption. Using N-containing, aromatic, ring-based Lewis basic ligands to establish MOFs is one main strategy for the pore engineering [20]. Yang et al. reported that the −NH_2_ functional group can exhibit a good effect and provide a strong affinity for CO_2_ molecules in adsorption [21]. At the same time, the procedure of introducing a functional group is complicated, and the pore window might be blocked by the functional groups [19,22].

The titanium-based MOF NH_2_–MIL125 (Material of Institute Lavoisier, MIL) has been investigated for different fields, such as gas storage, gas separation, and photocatalysis [23,24,25,26]. The amino-functionalization of MOFs is believed to improve CO_2_ adsorption, so NH_2_–MIL125 was chosen to be investigated. The structure of NH_2_–MIL125 is quasi-cubic tetragonal system, constructed with octameric Ti_8_O_8_(OH)_4_ oxo-clusters connected by amino-dicarboxylate linkers. In addition, the incorporation of alkali metal into Ti-MOF has not been reported. In this study, a sequence of *x*M@NH_2_–MIL125(Ti) (M = Li, Na, K) was successfully synthesized by in situ cation incorporation, in order to understand the impact of the doping of alkali metals on the CO_2_ adsorption capacity of NH_2_–MIL125(Ti).

## 2. Materials and Methods 

### 2.1. Chemicals

Titanium isopropoxide (98%, AR) and 2-aminoterephthalic acid (H_2_BDC–NH_2_, AR) were purchased from J&K Scientific Ltd. (Beijing, China). Lithium chloride (LiCl, AR), sodium chloride (NaCl, AR) and potassium chloride (KCl, AR) were purchased from Sinopharm Chemical Reagent Co., Ltd. (Shanghai, China). Methanol (CH_3_OH, AR) and *N*,*N*-dimethylformamide (DMF, AR) were provided by Tianjin Fuchen Chemicals Co. Ltd. (Tianjin, China). All the chemicals were used as received, without further purification.

### 2.2. Synthesis of NH_2_–MIL125(Ti)

The synthesis of NH_2_–MIL125(Ti) was performed by dissolving H_2_BDC–NH_2_ (1.449 g, 8 mmol) in 30 mL DMF/methanol (1:1 *V/V*) and stirred for 10 min at room temperature; then, titanium isopropoxide (1.308 g, 1.39 mL, 4.5 mmol) was added to the above solution and stirred for another 30 min. The mixture was transferred into a Teflon liner inserted in a stainless-steel autoclave. Then the autoclave was sealed and the mixture was heated for 16 h at 150 °C. After cooling slowly to ambient temperature, the obtained yellow powder was filtered and washed with DMF and methanol, and the resultant product was finally vacuum-dried at 60 °C overnight.

### 2.3. Synthesis of xM@NH_2_–MIL125(Ti)

The alkali metal-doped NH_2_–MIL125(Ti) samples were prepared through the in situ synthesis method. MCl (M = Li^+^, Na^+^, K^+^) was milled into fine particles and dissolved in DMF. The above solution containing M^+^ was then added to the solution containing H_2_BDC–NH_2_ and titanium isopropoxide. The synthesis and activation of the alkali metal cation incorporated NH_2_–MIL125(Ti) followed the same route of NH_2_–MIL125(Ti). The yellow powder product was denoted as *x*M@NH_2_–MIL125(Ti), where M is related to the alkali metal cation (Li^+^, Na^+^, K^+^), and *x* represents the mass ratio (1% and 2%) of alkali metal chloride to the pure NH_2_–MIL125(Ti). For the 1% case, as the sum mass of H_2_BDC–NH_2_ and titanium isopropoxide were 2.757 g, so 0.028 g of MCl (0.65 mmol for LiCl, 0.47 mmol for NaCl, and 0.37 mmol for KCl) was added. All the obtained samples after final filtration were vacuum-dried at 60 °C overnight. The yield, 90% of the product, is nearly the same as the pristine NH_2_–MIL125(Ti).

### 2.4. Sample Characterization

FT-IR spectra were obtained on AXS TENSOR-27 FT-IR spectrometer (Bruker, Karlsruhe, Germany), with KBr pellets at room temperature in the range of 4000–500 cm^−1^, with a resolution of 4 cm^−1^. Powder X-ray diffraction (PXRD) data were performed on a Advance-D8 (Bruker, Karlsruhe, Germany) with Cu K*_α_* radiation operating at 40 kV and 40 mA, in the range of 5° < 2*θ* < 50°, with a step length of 0.02° (2*θ*). X-ray photoelectron spectrometer (XPS) data were collected on an Axis Ultra DLD (Kratos Analytical Ltd. of Shimadzu Corporation, Manchester, UK) with Al K_α_ (h*ν* = 1486.7 eV), operating at 15 kV and 10 mA. All curves have been charge-corrected to the main line of the carbon C1’s spectral component (C/C, C/H), set to 284.80 eV. Thermal stability was characterized via TA-SDT Q600 (TA Instruments, NewCastle, DE, USA) from room temperature to 900 °C, under a heating rate of 5 °C·min^−1^ in N_2_ atmosphere and with a flowing rate of 10 mL·min^−1^. The contents of the metal ions were analyzed by ICP-OES730 (Agilent, Santa Clara, CA, USA). The scanning electron microscope (SEM) and energy-dispersive spectroscopy (EDS) were carried out using a Hitachi SU8020 (Hitachi High-Technologies Corporation, Tokyo, Japan). The powder surface was gold that had been metalized previously. N_2_ and CO_2_ adsorption isotherms were obtained using Autosorb-1 (Quantachrome Instruments, Boynton Beach, FL, USA). The samples were pre-treated to remove excess water molecules and impurities at 150 °C for 8 h under vacuum, and the CO_2_ adsorption isotherm was tested at 273 and 293 K.

## 3. Results

### 3.1. FT-IR Analysis

**Figure 1:**
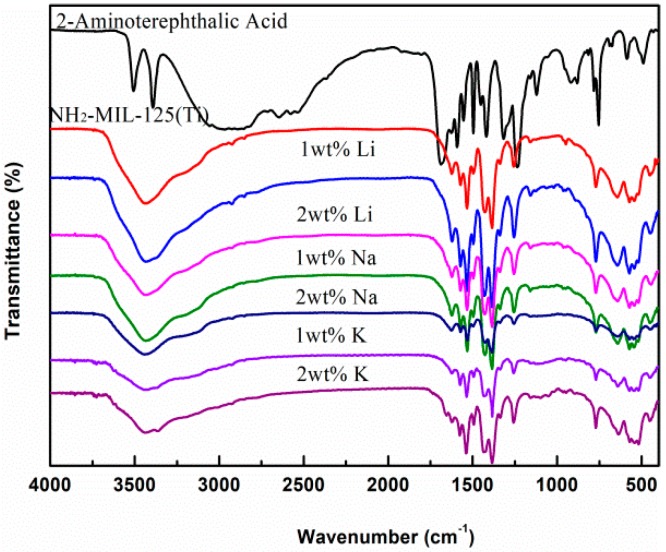
FT-IR spectra of H_2_BDC–NH_2_, NH_2_–MIL125(Ti) and *x*M@NH_2_–MIL125(Ti).

The FT-IR spectra of *x*M@NH_2_–MIL125(Ti), as shown in Figure 1, shows the characteristic vibration peaks before and after the alkali metal doping. There is no obvious change in the region of the peaks, but the intensity is slightly changed. To be specific, 3420–3452 cm^−1^ shows the contribution of −NH_2_ group in the structure. The vibration bands located at about 1637 and 1500 cm^−1^ belonged to carbonyl asymmetric stretching vibrations, and vibration bands located at 1298 cm^−1^ could be assigned to the C–H symmetric stretching vibrations of the benzene ring. The peak around 760 cm^−1^ is the Ti–O stretching for non-bound oxygen atoms, and 500–755 cm^−1^ is the Ti–O–Ti stretching vibration [27].

### 3.2. X-ray Diffraction Analysis

**Figure 2:**
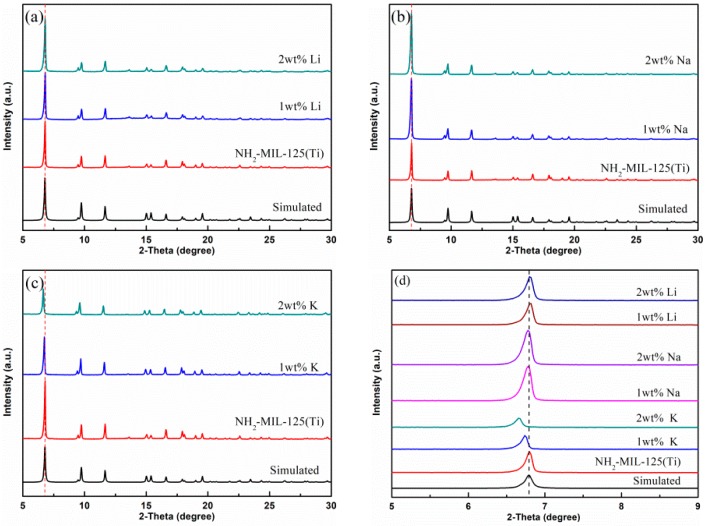
XRD patterns of doped NH_2_–MIL125(Ti): (**a**) *x*Li@NH_2_–MIL125(Ti), (**b**) *x*Na@NH_2_–MIL125(Ti), (**c**) *x*K@NH_2_–MIL125(Ti), and (**d**) magnification part for *x*M@NH_2_–MIL125(Ti).

XRD patterns of NH_2_–MIL125(Ti) synthesized with different loadings of Li, Na, and K are shown in Figure 2. It can be seen that all samples still maintained the crystal structure of NH_2_–MIL125(Ti). It shows that *x*M@NH_2_–MIL125(Ti) exhibited the distinct characteristic diffraction peaks at 2*θ* = 6.8°, 9.8°, 12.1°, 17.3°, and 18.8°, which were consistent with the experimental and calculated patterns of NH_2_–MIL125(Ti) [27], and no additional peaks were observed; this means that no other crystalline impurity phases formed after the modification, indicating that the crystal structure of NH_2_–MIL125(Ti) was well maintained. Therefore, we speculate that the doping of alkali metal ions during the solvothermal synthesis would not affect the crystalline structures of NH_2_–MIL125(Ti). However, the characteristic diffraction peak positions around 6.8° of *x*K@NH_2_-MIL125(Ti) have been shifted slightly for the case of potassium ion doping, as shown in Figure 2d, which is owing to the larger ionic radius of K^+^ than of Li^+^ and Na^+^ [28]. The larger ionic radius may cause differences in the construction of the framework during the solvothermal synthesis.

### 3.3. X-ray Photoelectron Spectrometer

**Figure 3:**
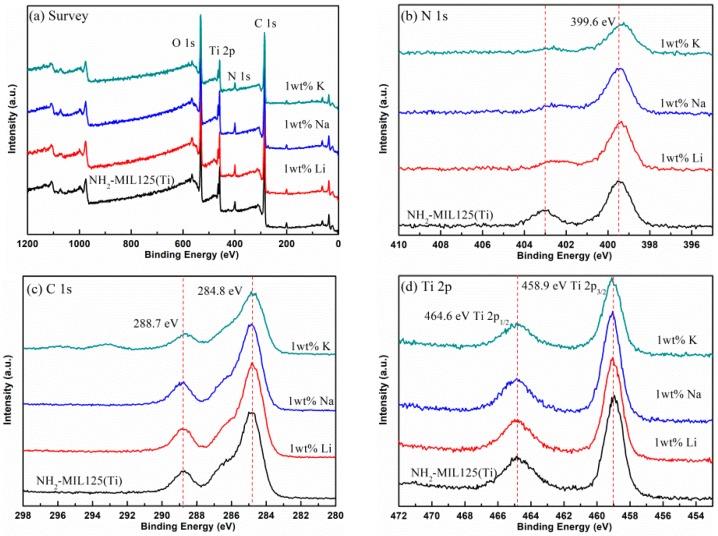
The X-ray photoelectron spectrometer (XPS) spectra of doped and pristine NH_2_–MIL125(Ti): (**a**) survey, (**b**) N 1s, (**c**) C 1s, (**d**) Ti 2p.

XPS measurements were carried out to determine the chemical composition and electronic structure of *x*M@NH_2_–MIL125(Ti). As shown in Figure 3, the wide-scan XPS spectra of all the samples show four peaks. The peak of 288.7 eV, corresponding to C 1s, indicates the existence of a C element. Similarly, the peaks of 399.6 eV for N at 1s, 458.9 eV for Ti at 2p, and 288.7 eV for O at 1s, indicate the existence of N, Ti, and O elements. In the 1s spectrum for N, the peak at 399.6 eV belonged to the N of the amine group protruding or stretching out into the cavities. The peak at 403.1 eV can be assigned to the positively charged –N=^+^ and –NH–^+^ [29]. The symmetric peaks in the Ti 2p spectrum located at 458.9 eV and 464.6 eV are attributed for Ti 2p_3/2_ and Ti 2p_1/2_, respectively. This means that the oxidation state Ti in the titanium–oxo cluster remains in IV. Combining with the experimental results of XRD and N_2_ adsorption, it can be inferred that the amine groups are not coordinated with metal ions, but may be protruding into the empty space of the internal porous [29]. The XPS peaks of *x*M@NH_2_–MIL125(Ti) are nearly the same as those of NH_2_-MIL125(Ti), which indicates that the alkali metal doping procedure has little influence on the electronic structures of NH_2_-MIL125(Ti).

### 3.4. Thermal Stability

**Figure 4:**
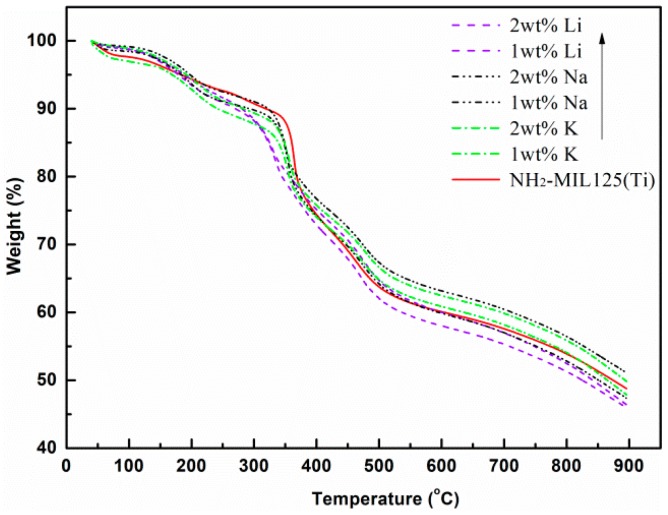
Thermogravimetric analysis (TGA) curves of NH_2_–MIL125(Ti) and *x*M@NH_2_–MIL125(Ti).

The thermal stability and decomposition temperatures of NH_2_–MIL125 and *x*M@NH_2_–MIL125 were investigated via thermogravimetric analysis (TGA). It can be seen from Figure 4 that there are three stages of weight losses in the TGA curves. NH_2_–MIL125, *x*Li@NH_2_–MIL125, *x*Na@NH_2_–MIL125, and *x*K@NH_2_–MIL125 showed similar weight-loss behaviors. The first weight loss, between 30 and 130 °C, was caused by the removal of physically adsorbed water, bound water, and free solvent molecules (such as methanol) [27]. At the region of 130 to 300 °C, the weight loss curves of all the samples gradually decreased, which was due to the removal of the DMF and non-coordinated organic linkers of H_2_BDC–NH_2_ trapped in the material. When the temperature reached around 300 °C, a sudden weight loss occurred, which corresponds to the degradation of the framework to TiO_2_ anatase [11,23]. The thermogravimetric results show that *x*M@NH_2_–MIL125 can remain thermally stable at 300 °C. 

### 3.5. Scanning Electron Microscopy

**Figure 5:**
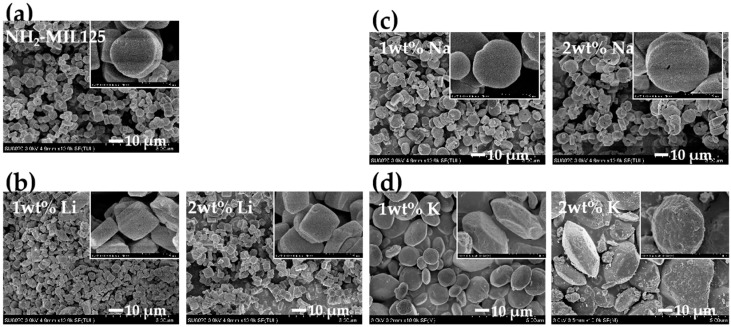
Scanning electron microscopy (SEM) images of doped and pristine NH_2_–MIL125(Ti): (**a**) NH_2_–MIL125(Ti), (**b**) *x*Li@NH_2_–MIL125(Ti), (**c**) *x*Na@NH_2_–MIL125(Ti), and (**d**) *x*K@NH_2_–MIL125(Ti).

The morphology of pristine NH_2_–MIL125 and *x*M@NH_2_–MIL125 are shown in Figure 5. NH_2_–MIL125 (Figure 5a) and *x*Na@NH_2_–MIL125 (Figure 5c) exert thin and circular plate shapes. The NH_2–_MIL125 involves an average particle size of 6 μm, which is similar to the conventional synthetic method of NH_2_–MIL125 (5 μm) [23], while *x*Li@NH_2–_MIL125 has a tetragonal plate shape with a particle size of 5 μm (Figure 5b). The average particle size of *x*Na@NH_2_–MIL125 is 8 μm, with a circular plate shape (Figure 5c). However, *x*K@NH_2_–MIL125 has a truncated octahedron shape with the particle size of 15 μm (Figure 5d). The crystal size increases in the sequence of Li, Na, and K, reflecting the different growth rates of the *x*M@NH_2_–MIL125. The titanium precursor contains different amount of alkali metal ions during the solvothermal synthesis. As for the case of 1M@NH_2_–MIL125(Ti), there was 0.65 mmol of LiCl, 0.47 mmol of NaCl, and 0.37 mmol of KCl in the solvothermal precursor, respectively. The titanium precursor containing K ions involves the lowest doping ions molar concentration, which may have less influence on the moving of Ti and the growing rate of Ti–O in the titanium precursor, resulting in the highest particle size.

The alkali metal incorporation has an influence on the kinetics of hydrolysis and condensation reactions. The morphology evolution of the crystal has affected the changes of NH_2_–MIL125. Thus, special polyhedrons of NH_2_-MIL125 from the tetragonal plate through the circular plate to a truncated octahedron were obtained. The crystal morphology of NH_2_–MIL125(Ti) changing in our work is a little different from what Hu et al. previously reported [27]. They controlled the crystal morphology from the circular plate to the octahedron, by altering the concentration of reactants from low to high and by changing the total solvent volume. In our work, the concentration of the reactant was the same when adding different kind of alkali metal salts, but the total ion concentration was decreased in the sequence of Li, Na, and K, which caused the different growth rates. Hu et al. reported that the perfect NH_2_–MIL125 crystal should be rhombic dodecahedron morphology by Bravais-Friedel-Donnay-Harker (BFDH) theory [30]. It can be concluded that these morphology changes were affected by the total concentration of the ions in the solvothermal precursors. 

**Figure 6:**
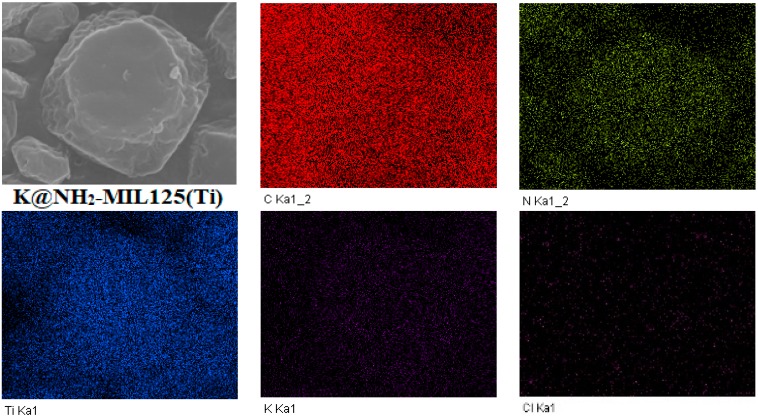
SEM–energy-dispersive spectroscopy (EDS) mapping of 1K@NH_2_–MIL125(Ti) elements: C, N, Ti, K, and Cl.

To verify that the alkali metals were doped into NH_2_–MIL125 homogeneously, the mapping images of the 1K@NH_2_–MIL125 through SEM–EDS were performed. As can be seen in Figure 6, the C, N, Ti, K, and Cl elements are uniformly dispersed in the 1K@NH_2_–MIL125, indicating that the K ions have been well distributed into the framework of the 1K@NH_2_–MIL125. The same results were found in the 1Na@NH_2_–MIL125(Ti). The doped alkali metal ions might be well incorporated into the structure, or just dispersed on the surface of the Ti_8_O_8_(OH)_4_–(O_2_C–C_6_H_5_–CO_2_–NH_2_)_6_. The alkali metals presumably exist in the form of chlorides, because the alkali oxide needs a higher temperature to be obtained [31]. The signal intensity of lithium is too weak to determine its dispersion in *x*Li@NH_2_–MIL125 by SEM–EDS imaging, due to the high background noises.

### 3.6. Alkali Metal Content

Inductively Coupled Plasma-Optical Eemission Spectroscopy (ICP–OES) analyses were performed to examine the presence and quantity of the alkali metal for xLi@NH_2_–MIL125(Ti), and the results are listed in Table 1. The experimental titanium content for both doped and original NH_2_–MIL125(Ti) are nearly the same, which is between 15.7~16.9 wt %. This is close to the theoretical calculating value 23 wt % for NH_2_–MIL125(Ti) and Ti_8_O_8_(OH)_4_–(O_2_C–C_6_H_3_–CO_2_–NH_2_)_6_ [11]. The higher lithium content can be observed by increasing the lithium loading in the precursor. The amount of Li and Na in the framework drops greatly after a series of solvent exchanging treatment, because the alkali metal does not coordinate with the organic ligands and its small atomic radius. For the K case, maybe there are more K ions trapped in the pores or on the surface of the framework, so the ICP results of K are higher than those for Li and Na.

### 3.7. N_2_ Asorption Isotherms

**Figure 7:**
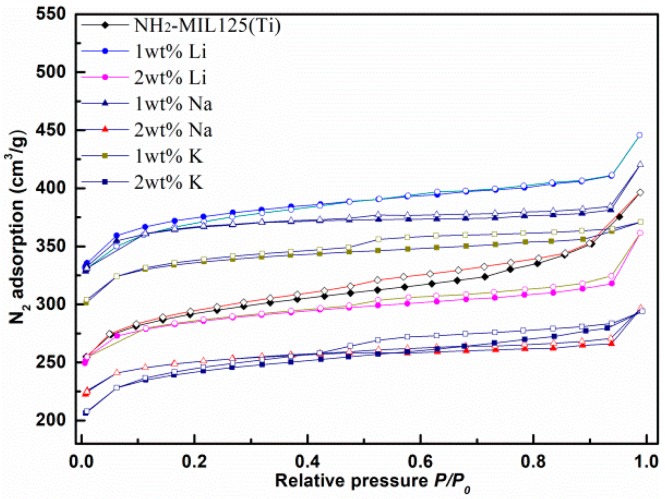
N_2_ adsorption isotherms at 77 K for NH_2_–MIL125(Ti) and *x*M@NH_2_–MIL125(Ti).

Nitrogen adsorption isotherms were measured to obtain the porosity of *x*M@NH_2_–MIL125, and the results are shown in Figure 7. All obtained N_2_ adsorption isotherms of the *x*M@NH_2_–MIL125 series exhibited a type I isotherm curve. The Brunauere Emmette Teller (BET) surface area was calculated in the relative pressure (P/P_0_) range of 0.05 to 0.3, and the detailed adsorption data were summarized in Table 1. The NH_2_–MIL125(Ti) structure involves octahedral cages of 12.5 Å and tetrahedral cages of 6.1 Å, and are accessible through triangular windows of 5–7 Å. At low relative pressure, the significant adsorption amount indicates that the materials involve microporosity [22,28].

It was found that the surface areas of *x*M@NH_2_–MIL125 increased observably after alkali cation doping, in the sequence of 1Li^+^ > 1Na^+^ > 1K^+^ > 2Li^+^ > 2Na^+^ > 2K^+^. The surface areas are 1470, 1451, and 1226 m^2^·g^−1^ after 1 wt % Li, Na, K incorporation, respectively, which is a significant improvement compared with 1038 m^2^·g^−1^ for the pristine NH_2_–MIL125(Ti). It is worth pointing out that the BET specific surface area of the pristine NH_2_–MIL125(Ti) in our work is lower than those reported in the reference, including 862~1469 m^2^ g^−1^ [23], 1203 m^2^ g^−1^ [21], 1215 m^2^ g^−1^ [32], and 1041~1268 m^2^ g^−1^ [27]. The pristine NH_2_–MIL125(Ti) and the *x*M@NH_2_-MIL125 are using the same synthetic procedure and calcination temperatures in this work, to investigate the tendency of the alkali metal doping effects. 

The enhancement of 1Li@NH_2_–MIL125 was greater than that observed for 1Na@NH_2_–MIL125 and 1K@NH_2_–MIL125. The increasing trend of surface area correlates excellently with the conclusions from the SEM and XRD investigations, as described previously. However, the specific surface area dropped rapidly when the alkali metal doping amount increased to 2 wt %. Perhaps a doping amount 2 wt % of the dopants themselves (Li, Na, and K) is too much, and the channels or pores of NH_2_-MIL125 might be blocked [8]. Therefore, the alkali metal doping can promote the specific surface area and pore volumes of the NH_2_–MIL125(Ti), but excessive doping will block the pores of metal organic framework, consequently reducing its performance. It is very important to select suitable alkali metal type and doping dosage for the purpose of improving the gas adsorption performance of MOFs.

### 3.8. CO_2_ Adsorption Isotherms Measured

**Figure 8:**
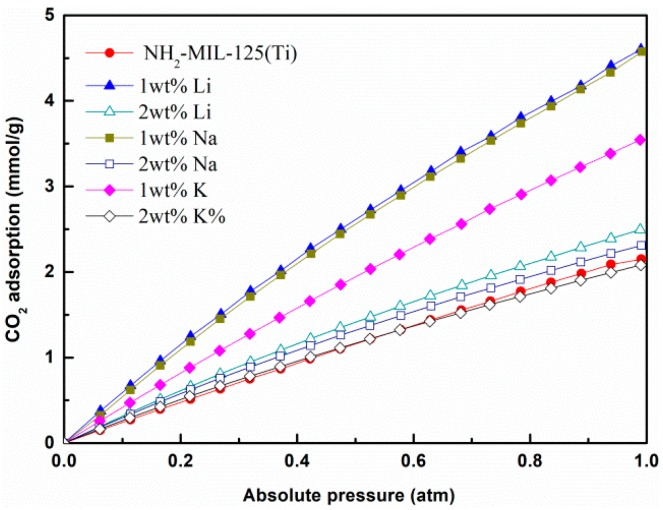
CO_2_ adsorption isotherms of NH_2_–MIL125(Ti) and *x*M@NH_2_–MIL125(Ti) at 293 K.

CO_2_ adsorption isotherms of NH_2_–MIL125(Ti) and *x*M@NH_2_–MIL125(Ti) at 293 K and 1 atm are shown in Figure 8. All the samples show a steep initial increase at low pressures, which is characteristic of a high-CO_2_-adsorption material with microporsity. In the research system for carbon dioxide adsorption, the presence of amino functionalization provides more active sites, NH_2_–MIL125 showed a higher quantity of CO_2_ adsorption, as summarized in Table 2. 

It can be seen from the CO_2_ adsorption isotherms that the CO_2_ adsorption amount of *x*M@NH_2_–MIL125(Ti) increased when the 1 wt % alkali metal was introduced during the solvothermal synthesis. The adsorption amounts are 4.60, 4.57, and 3.55 mmol g^−1^ for 1Li@NH_2_–MIL125(Ti), 1Na@NH_2_–MIL125(Ti), and 1K@NH_2_–MIL125(Ti), respectively. The CO_2_ adsorption capacity of 1M@NH_2_–MIL125 is in the sequence of Li > Na > K, which is consistent with the order of the decreasing ionic radius, and in agreement with the N_2_ adsorption performance. Of these compounds, 1Li@NH_2_-MIL125(Ti) showed the highest CO_2_ adsorption capacity, but the amount is still much lower than that of Mg-MOF-74 [36]. 

**Figure 9:**
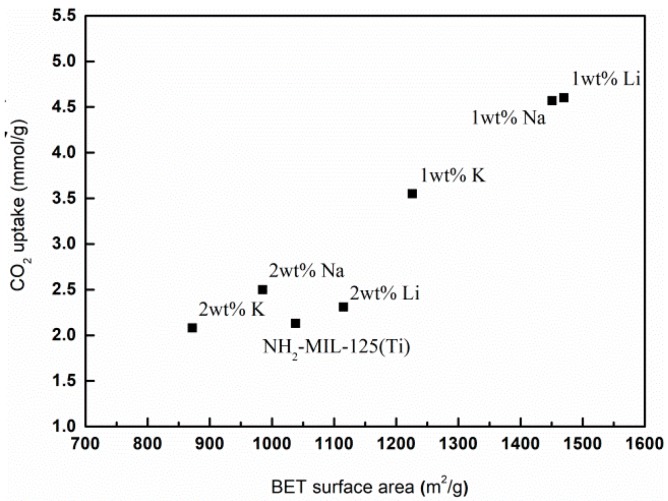
The relationship of CO_2_ uptake on *x*M@NH_2_–MIL125(Ti) with *S_BET_* at 293 K and 1 atm.

One reason why alkali metal doping increases the carbon dioxide adsorption capacity is the influence of the framework structure. The enhancement is maybe due to the defect site of the frameworks caused by the alkali metal doping tuning the crystallite framework (interpenetrated level), surface (surface area, pore etc.), and adsorptive site (dipole interaction) [28]. As we discussed before, the alkali metal doped *x*M@NH_2_–MIL125(Ti) has a larger specific surface area, and the morphology of the MOFs changed. The specific surface area of NH_2_–MIL125(Ti) increases due to the decrease of the particle size, which leads to a large amount of CO_2_ adsorption. The CO_2_ adsorption capacity has a positive correlation with the S*_BET_*, as shown in Figure 9. The surface area of the 1Li@NH_2_–MIL125(Ti) is much higher than that of the NH_2_–MIL125(Ti) and 2Li@NH_2_–MIL125(Ti), so the higher CO_2_ adsorption of 1Li@NH_2_–MIL125(Ti) is understandable. The approximate CO_2_ adsorption capacity for 1Li@NH_2_–MIL125(Ti) and 1Na@NH_2_–MIL125(Ti) may because the surface area of them are closed.

The results of the CO_2_ adsorption on *x*M@NH_2–_MIL125(Ti) is not only relevant to the specific surface area, but also to the alkali metal dispersed in the framework of the *x*M@NH_2_–MIL125(Ti). Another reason why alkali metal doping increases the carbon dioxide adsorption capacity is that carbon dioxide preferentially adsorbs around the alkali metal ions, and computer simulations by Mu et al. confirm this view [16]. They found that the first ionization energies and the electron donating ability of the alkali metal atoms are much lower than the boron group metal and alkaline earth metal atoms, thereby causing more ion partial charges. The first ionization energy is the minimal energy required to remove an electron from the atom or molecule isolated in free space and in its ground electronic state. The electrostatic interaction between CO_2_ and MOFs increases with increasingly induced charges. The 2Na@NH_2_–MIL125(Ti) has a bit of a smaller surface area than NH_2_–MIL125(Ti), but a larger CO_2_ adsorption, which may contribute to its smaller pore size and the doping of alkali metals. Therefore, the doping of alkali metal helps to improve the CO_2_ adsorption properties of NH_2_-MIL125(Ti).

**Figure 10:**
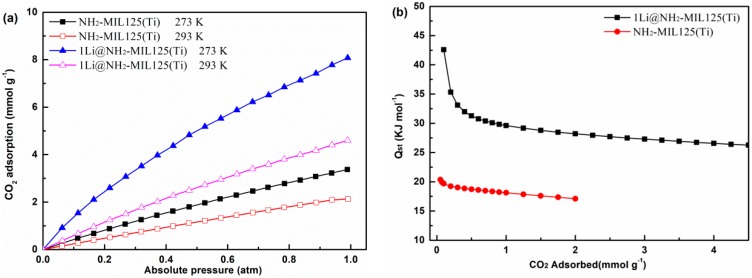
(**a**) The CO_2_ adsorption isotherms at 273 and 293 K. (**b**) The heats of CO_2_ adsorption of 1Li@NH_2_–MIL125 and NH_2_–MIL125.

The heat of CO_2_ adsorption for NH_2_–MIL125 and 1Li@NH_2_–MIL125 were calculated using the Clausius–Clapeyron equation, based on the CO_2_ adsorption isotherms at 273 and 293 K, as shown in Figure 10. Of those tested, 1Li@NH_2_–MIL125 showed a higher heat of CO_2_ adsorption (42–26 kJ mol^−1^) than NH_2_–MIL125 (21–17 kJ mol^−1^). However, the value is lower than that of MIL-100 (63–23 kJ mol^−1^) [23]. The heat of CO_2_ adsorption at a CO_2_ coverage of 2.0 wt % for 1Li@NH_2_–MIL125 is estimated to be 28.2 kJ mol^−1^, which is much higher than that of NH_2_–MIL125 (17.1 kJ mol^−1^), suggesting a much stronger interaction between adsorbed CO_2_ and 1Li@NH_2_–MIL125.

### 3.9. CO_2_ Adsorption Regenerability

**Figure 11:**
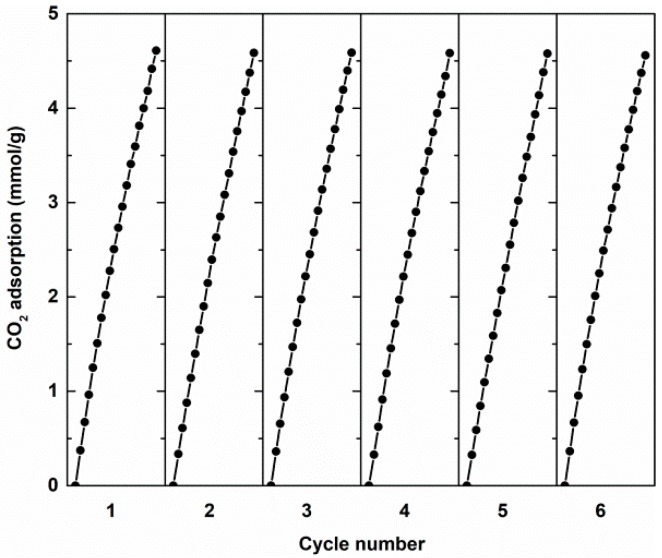
Recycle test of CO_2_ adsorption on 1Li@NH_2_–MIL125(Ti) at 293 K.

The recycle test of 1Li@NH_2_–MIL125(Ti) on CO_2_ adsorption was performed to test its regenerability for gas capture applications, because of it having the highest capacity for CO_2_ adsorption in *x*M@NH_2_–MIL125(Ti). Figure 11 shows the static CO_2_ adsorption capacity of 1Li@NH_2_-MIL125(Ti) at 293 K. The sample was desorpted by vacuum before the new test. The readsorption amount is nearly stable for six cycles, with no obvious loss in activity. This indicates that the adsorption mechanism of CO_2_ is mainly based on physical adsorption [8].

## 4. Conclusions

A series of *x*M@NH_2_–MIL125(Ti) has been synthesized by in situ incorporation of alkali metal, which is straightforward and can tune the morphology of MOFs, compared with post-modification procedures. The effect of in situ alkali metal incorporation into the structure was demonstrated through the experimental data of N_2_ and CO_2_ adsorption of MOFs. The CO_2_ adsorption is mainly affected by the van der Waals forces between the framework and CO_2_ molecules. The smaller pore size, the Lewis basic active site −NH_2_ groups, and the alkali metals in our work are all beneficial for CO_2_ adsorption. The introduction of alkali metal mainly affects the CO_2_ adsorption in two respects. On the one hand, it impacts the crystal growth rate of MOFs, resulting the different morphology and size of grains, thus leading to the differences in specific surface area and pore size, which has a significant impact on the CO_2_ adsorption. On the other hand, the alkali metals, existing in the final framework after a series of solvent exchanging treatments, have an influence on the CO_2_ adsorption. This enhancement is due to the increased specific surface area and alkali metal coordinated on the defects of the cage construction. It was found that 1Li@NH_2_–MIL125(Ti) is the most efficient CO_2_ adsorbent among *x*M@NH_2_–MIL125(Ti). Although the amount is still lower than the reported MOFs, such as Mg-MOF-74, alkali metal doping has proven to be an effective modification strategy. This preliminary study is a practical attempt to improve the performance of MOF materials on CO_2_ adsorption, which will promote related research on CO_2_ conversion reactions.

## Figures and Tables

**Table 1:** Textural properties of NH_2_–MIL125 doped with different amounts of alkali metal.

Samples	*S*_BET_ (m^2^ g^−1^)	CO_2_ Adsorption (mmol g^−^^1^)	Alkali Metal Introduced in the Synthesis (wt %)	Metal Analysis (ICP, wt %)	
				Ti	Alkali Metal
NH_2_–MIL125(Ti)	1038	2.13	0	16.3	0
1Li@NH_2_–MIL125(Ti)	1470	4.60	0.164	16.7	0.082
2Li@NH_2_–MIL125(Ti)	1115	2.31	0.327	16.1	0.099
1Na@NH_2_–MIL125(Ti)	1451	4.57	0.393	15.7	0.065
2Na@NH_2_–MIL125(Ti)	985	2.50	0.787	16.9	0.118
1K@NH_2_–MIL125(Ti)	1226	3.55	0.524	15.6	0.378
2K@NH_2_–MIL125(Ti)	872	2.08	1.049	15.8	0.456

**Table 2:** Comparison of CO_2_ adsorption on reported metal–organic frameworks (MOFs).

MOFs	Condition	CO_2_ Adsorption (mmol g^−1^)	References
PEHA-MIL-101	298 K, 10 bar	1.30	[33]
MIL-101(Cr)	298 K, 10 bar	0.85	[33]
NH_2_-UiO-66	298 K, 1 bar	3.15	[34]
UiO-66-AD6	298 K, 1 bar	2.63	[35]
Mg-MOF-74	298 K, 1 bar	7.95	[36]
MIL125(Ti)	298 K, 1 bar	3.00	[11]
NH_2_-MIL125(Ti)	298 K, 1 bar	2.18	[11]
NH_2_-MIL125(Ti)	293 K, 1 bar	2.13	This work
1Li@NH_2_-MIL125(Ti)	293 K, 1 bar	4.60	This work
1Na@NH_2_-MIL125(Ti)	293 K, 1 bar	4.57	This work
1K@NH_2_-MIL125(Ti)	293 K, 1 bar	3.55	This work
